# Ideal Cardiovascular Health in Young Adults With Established Cardiovascular Diseases

**DOI:** 10.3389/fcvm.2022.814610

**Published:** 2022-02-17

**Authors:** Jane A. Leopold, Elliott M. Antman

**Affiliations:** Division of Cardiovascular Medicine, Brigham and Women's Hospital, Harvard Medical School, Boston, MA, United States

**Keywords:** cardiovascular disease in the young, ideal cardiovascular health, surveys, digital health devices, direct to participant research

## Abstract

There has been an increase in the prevalence of cardiovascular diseases among young adults in the United States that has been attributed, in part, to a rise in overweight and obesity, use of combustible tobacco and unhealthy diet and exercise patterns. These factors are influenced further by socioeconomic status and other social determinants of health. In the My Research Legacy study, we examined ideal cardiovascular health in young adults aged 18– <50 years with cardiovascular disease using the Life's Simple 7 survey and data from digital health devices. Young adults with cardiovascular disease (*n* = 349) were older, had a lower socioeconomic status, a higher prevalence of risk factors, and lower Life's Simple 7 Health Scores (6.4 ± 1.5 vs. 7.1 ± 1.5, *p* < 0.01) compared to young adults without cardiovascular disease (*n* = 696). Analysis of digital health device data revealed that young adults with cardiovascular disease performed a similar number of weekly minutes of moderate and vigorous exercise as those without disease leading to similar ideal activity scores. Young adults with cardiovascular disease also shared similarities in modifiable risk factors with adults aged ≥50 years with cardiovascular disease (*n* = 217), including weight, dietary habits, and weekly minutes of exercise. Latent class analysis identified two phenogroups of young adults with cardiovascular disease: phenogroup 1 was characterized by more advantageous cardiovascular health factors and behaviors resulting in higher Life's Simple 7 Health Scores than phenogroup 2 (7.4 ± 1.2 vs. 5.5 ± 1.1, *p* < 0.01). These findings in young adults with cardiovascular disease may inform the design of behavioral and therapeutic interventions in the future to decrease cardiovascular morbidity and mortality.

## Introduction

Although there has been a significant decline in mortality attributable to cardiovascular diseases, there is accumulating evidence that this benefit may be age-specific and less applicable to younger patients (1, 2). Reports have noted an increasing prevalence of cardiovascular diseases in individuals under the age of 55 years, including higher rates of myocardial infarction and stroke (3–5). The etiology of the rise in cardiovascular diseases in younger adults is multifactorial and has occurred concomitant with an increase in the proportion of populations that are overweight and obese; have prevalent hypertension, hyperlipidemia, and diabetes mellitus; continue to use combustible tobacco and e-cigarette products; and consume highly processed foods (6–9). These health factors and habits are influenced further by racial and ethnic, socioeconomic, and gender disparities resulting in a higher incidence and prevalence of cardiovascular disease in select community-based populations (7, 9, 10).

The American Heart Association has prioritized achieving ideal cardiovascular health for all as a 2,030 strategic impact goal with a focus on increasing health equity among populations, overall community, and individual well-being, and decreasing cardiovascular mortality (11, 12). The American Heart Association assesses ideal cardiovascular health as a composite of modifiable health behaviors (exercise, diet, tobacco use) and health factors (weight, blood pressure, cholesterol, fasting blood glucose) (13). The importance of achieving and maintaining ideal cardiovascular health has been confirmed by observational studies that associated it with a lower incidence of cardiovascular disease and mortality (14–18). Despite this, ideal cardiovascular health has declined over the past 20 years in communities (16, 18) and an analysis from the National Health and Nutrition Examination Survey (NHANES) estimated that ~70% of all cardiovascular disease events were related to non-ideal cardiovascular health (19).

Among young adults aged 20–49 years in NHANES, analysis of ideal cardiovascular health habits and behaviors reported that the percentage of individuals with non-ideal scores for BMI, physical activity, or healthy diet was ≥50%. Although the percentage of individuals with non-ideal scores for smoking, cholesterol, blood pressure, or fasting blood glucose levels was ≤ 45%, there was no single Life's Simple 7 health factors or habits category where all individuals achieved an ideal score (20). In the current study, we examined ideal cardiovascular health in young adult participants (age <50 years) in the American Heart Association's My Research Legacy study. Since our study sample was enriched for individuals with established cardiovascular disease, we hypothesized that young adults with cardiovascular diseases would represent a heterogeneous group with differences in ideal cardiovascular health factors and habits compared to young adults without cardiovascular disease.

## Methods

### Study Cohort

The My Research Legacy study (NCT 02958098) was a direct-to-participant study sponsored by the American Heart Association that was open for enrollment between November 2016 and October 2018 (21). The study was approved by the Advarra Institutional Review Board (#31995) and all participants signed informed consent. Participants were eligible for the study if they resided in the United States, were ≥18 years old and had internet access. Baseline demographics were self-reported. History of cardiovascular disease was broadly defined as prior myocardial infarction, stroke, atrial fibrillation, systolic heart failure, cardiomyopathy, and aortic dissection. Participants were considered to have prior cardiovascular disease if they responded affirmatively to a series of disease-specific questions that were derived by a panel of subject matter experts (Supplementary Table 1). The affluence index, a measure of socioeconomic status, was derived from the National Neighborhood Data Archive (https:openicpsr.org), which is based on zip code tabulation areas. This validated index ranges from 0 to 1.0 and is defined by an average of census indicators (college education, incomes >$75K, and managerial and professional occupations) (22). A zip code crosswalk was utilized to align the affluence index with participant zip codes (23).

### Ideal Cardiovascular Health Assessment

Ideal cardiovascular health was assessed using the Life's Simple 7 survey (11, 13). This instrument includes questions related to cardiovascular health-related factors (weight, blood glucose, total cholesterol, and blood pressure levels) and behaviors (diet, exercise, combustible tobacco use). Ideal cardiovascular health is assessed by assigning a score of 0, 1, or 2 (poor, intermediate, or ideal, respectively) for each of the 7 cardiovascular health and habit categories according to prespecified criteria. A final Health Score that incorporates medication used to manage cardiovascular disease risk factors is calculated and can range between 0 (poor) and 10 (ideal). A state of ideal cardiovascular health was defined by having an overall Health Score of >7.0 or by meeting ideal criteria (category score = 2) for a minimum of 5 categories (12, 24).

### Digital Health Device Data

A subset of participants either registered their own digital health device with the study or were provided with a Fitbit Charge 2 device (25). Individuals with digital scales linked them to their devices to capture weight data. Digital health data were uploaded in 24-h increments to Validic (Validic Inc., Durham, NC). Deidentified data were parsed into weight, fitness, and routine categories using device-specific algorithms via the Validic API and transmitted to secure severs that were managed by The Broad Institute (Cambridge, MA) and REAN Cloud LLC (Herndon, VA).

### Latent Class Analysis

A series of latent class models were fitted using the Life's Simple 7 health and habit data for participants aged <50 years with cardiovascular disease. Latent class analysis is a probabilistic clustering methodology that assumes that within a population there are latent or unmeasured classes and that the relationship between any two variables is explainable by the latent variables (26). This model-based clustering approach has been used to identify clusters of individuals with cardiovascular disease and assign an individual to a class (or cluster) where that individual has the greatest probability of membership (27–30). Latent class analysis uses item-response probability, which is the probability that a variable is conditional for class membership, to identify the distinctness of each class (28–30). Model fitting is performed using the maximum likelihood method and several models are generated to determine the model with the optimum number of classes (or clusters) that adequately describes the dataset. Analyses are conducted in a stepwise manner starting with a 1-class model and the number of classes is increased with each iteration (30). The optimum number of classes was based on the criteria for model selection, which included the Bayesian Information Criteria, Vuong-Lo-Mendell-Rubin likelihood ratio test and class size. Following model selection, participants were assigned to their class based on posterior probability (26). The analyses were performed using MPlus (version 8.6, Muthén & Muthén, Los Angeles, CA).

### Sample Size Calculation and Statistical Analysis

The sample size to ensure that young adults with cardiovascular disease were represented adequately in the analyses was calculated based on the study enrollment of 1,561 participants with complete Life's Simple 7 survey data. With a prevalence of cardiovascular diseases of 40% in young adults aged <50 years (24), to achieve 95% power with an alpha = 0.05, the minimum sample size of young adults with cardiovascular disease required was 299 participants. For older adults aged ≥50 years, with a prevalence of cardiovascular diseases of 70% (24) to achieve 90% power with an alpha = 0.05, the minimum sample size required was 199 participants.

Data are presented as mean ± SD and *p*-values < 0.05 were considered significant. Normality of the data was tested using the Shapiro-Wilk test. Comparisons between continuous variables with normal distributions were analyzed using *t*-tests. Comparisons between categorical variables were analyzed using the chi-square test or Fisher's exact test as applicable. Non-parametric testing was done using the Wilcoxon-rank sum test. Propensity score matching was performed to compare young adults with prior myocardial infarction or cardiomyopathy/systolic heart failure to young adults without cardiovascular disease who had similar characteristics. Propensity score matching was done using a logit model that was adjusted for age, gender, race and ethnicity, and region at a ratio of 1:1. Data were analyzed using Stata 15/SE 15.1 (StataCorp LLC, College Station, TX) and Prism 9.0 (GraphPad, San Diego, CA).

## Results

### Ideal Cardiovascular Health in Young Adults With vs. Without Cardiovascular Disease

The My Research Legacy study enrolled 1,045 individuals aged <50 years of whom 349 had prior cardiovascular disease and 696 had no prior history of cardiovascular disease. Among the 349 individuals with established cardiovascular disease, 63 participants had a prior stroke, 130 participants had a prior myocardial infarction, 107 participants had cardiomyopathy and/or systolic heart failure, 13 had an aortic dissection, and 52 had atrial fibrillation. Sixteen participants had more than one diagnosis.

Young adults with cardiovascular disease were slightly older (39.8 ± 6.7 vs. 35.3 ± 8.4 yrs, *p* < 0.01), more likely to be Black, Asian, or Hispanic race and ethnicity and had a lower affluence index compared to individuals without cardiovascular disease (Table 1). Participants with cardiovascular disease were more likely to be current or prior smokers and had a higher prevalence of diabetes mellitus, hypertension, and hypercholesterolemia as well as medication use for these comorbidities compared to individuals without cardiovascular disease (all *p* < 0.01). Weight and body mass index (31.4 ± 8.7 vs. 29.3 ± 8.0 kg/m^2^, *p* < 0.01) and blood glucose levels (*p* < 0.01) were higher in young adults with cardiovascular disease compared to those without disease, but total cholesterol levels were lower (*p* < 0.05).

**Table 1:** Self-reported demographics, clinical, and Life's Simple 7 data for participants age <50 yrs.

	**Age <50 years No CVD (*n* = 696)**	**Age <50 yrs CVD (*n* = 349)**	***P*-value**
Age (yrs)	35.3 ± 8.4	39.8 ± 6.7	<0.01
Gender (no., % female)	572 (82.2)	293 (84.0)	0.48
**Race and ethnicity (no.)**			
Asian	26	5	<0.02
Black	20	21	
Hispanic	39	16	
White	590	290	
Other	21	17	
**Region (no.)**			
Northeast	104	59	0.31
South	264	147	
Midwest	175	75	
West	153	68	
Affluence status	0.43 ± 0.15	0.39 ± 0.14	<0.01
**ASCVD risk factors**			
Diabetes mellitus (no., %)	35 (5.0)	53 (15.2)	<0.01
Hypertension (no., %)	244 (35.1)	210 (60.2)	<0.01
Hypercholesterolemia (no., %)	234 (33.6)	201 (57.6)	<0.01
**Medications (no., %)**			
Diabetes mellitus	28 (4.0)	46 (13.2)	<0.01
Hypertension	99 (14.2)	167 (47.9)	<0.01
Hypercholesterolemia	32 (4.6)	101 (28.9)	<0.01
**Smoking status (no., %)**			
Current	49 (7.0)	38 (10.9)	<0.01
Quit ≤ 1 year	16 (2.3)	32 (9.2)	
Quit >1 year	124 (17.8)	106 (30.4)	
Never	507 (72.9)	173 (49.5)	
**Clinical data**			
Weight (kg)	82.5 ± 24.2	87.9 ± 26.1	<0.01
BMI (kg/m^2^)	29.3 ± 8.0	31.4 ± 8.7	<0.01
Systolic blood pressure (mmHg)*	116.4 ± 10.5	116.5 ± 14.9	0.98
Diastolic blood pressure (mmHg)*	73.3 ± 7.5	72.6 ± 10.2	0.25
Total cholesterol (mmol/L)*	187.9 ± 22.4	184.6 ± 29.1	<0.05
Blood glucose (mmol/L)*	95.5 ± 16.2	99.9 ± 20.8	<0.01
**Diet**			
Vegetables/day (cups)	1.8 ± 1.3	1.9 ± 1.3	0.79
Fruit/day (cups)	1.4 ± 1.1	1.4 ± 1.1	0.83
Fish (servings/week)	0.8 ± 1.0	0.9 ± 1.0	0.27
Whole grains (servings/day)	1.8 ± 1.3	1.5 ± 1.1	<0.01
Sugar-sweetened beverages (servings/week)	2.8 ± 3.5	2.7 ± 3.4	0.83
Avoid prepackaged foods (no., %)	311 (44.7)	193 (55.3)	<0.01
Avoid eating out (no., %)	212 (30.5)	167 (47.9)	<0.01
Avoid salt at home (no., %)	308 (44.3)	246 (70.5)	<0.01
**Exercise**			
Moderate exercise (min/week)	198.5 ± 211.9	215.5 ± 236.5	0.24
Vigorous exercise (min/week)	76.5 ± 118.1	53.8 ± 107.3	<0.01
**Life's Simple 7**			
**Smoking score (no., %)**			<0.01
Poor	49 (7.0)	38 (10.9)	
Intermediate	16 (2.3)	32 (9.2)	
Ideal	631 (90.7)	279 (79.9)	
**Activity score (no., %)**			0.08
Poor	14 (2.0)	7 (2.0)	
Intermediate	251 (36.1)	146 (41.8)	
Ideal	431 (61.9)	196 (56.2)	
**Diet score (no., %)**			<0.01
Poor	348 (50.0)	149 (42.7)	
Intermediate	303 (43.5)	163 (46.7)	
Ideal	45 (6.5)	37 (10.6)	
**Weight score (no., %)**			<0.01
Poor	270 (38.8)	172 (49.3)	
Intermediate	164 (23.6)	81 (23.2)	
Ideal	262 (37.6)	96 (27.5)	
**Blood glucose score (no., %)**			<0.01
Poor	16 (2.3)	23 (6.6)	
Intermediate	123 (17.7)	87 (24.9)	
Ideal	557 (80.0)	239 (68.5)	
**Cholesterol score (no., %)**			<0.01
Poor	14 (2.0)	5 (1.4)	
Intermediate	207 (29.7)	192 (55.0)	
Ideal	475 (68.3)	152 (43.6)	
**Blood pressure score (no., %)**			<0.01
Poor	29 (4.2)	25 (7.2)	
Intermediate	261 (37.5)	197 (56.5)	
Ideal	406 (58.3)	127 (36.4)	
**LS7 Health Score**	7.1 ± 1.5	6.4 ± 1.5	<0.01

*Contains imputed data from Life's Simple 7. Categorical variables are analyzed by Chi-Square test. Continuous variables are analyzed by t-test. Non-parametric variables were analyzed by Wilcoxon rank-sum test. CVD, cardiovascular disease; ASCVD, atherosclerotic cardiovascular disease.

**Figure 1:**
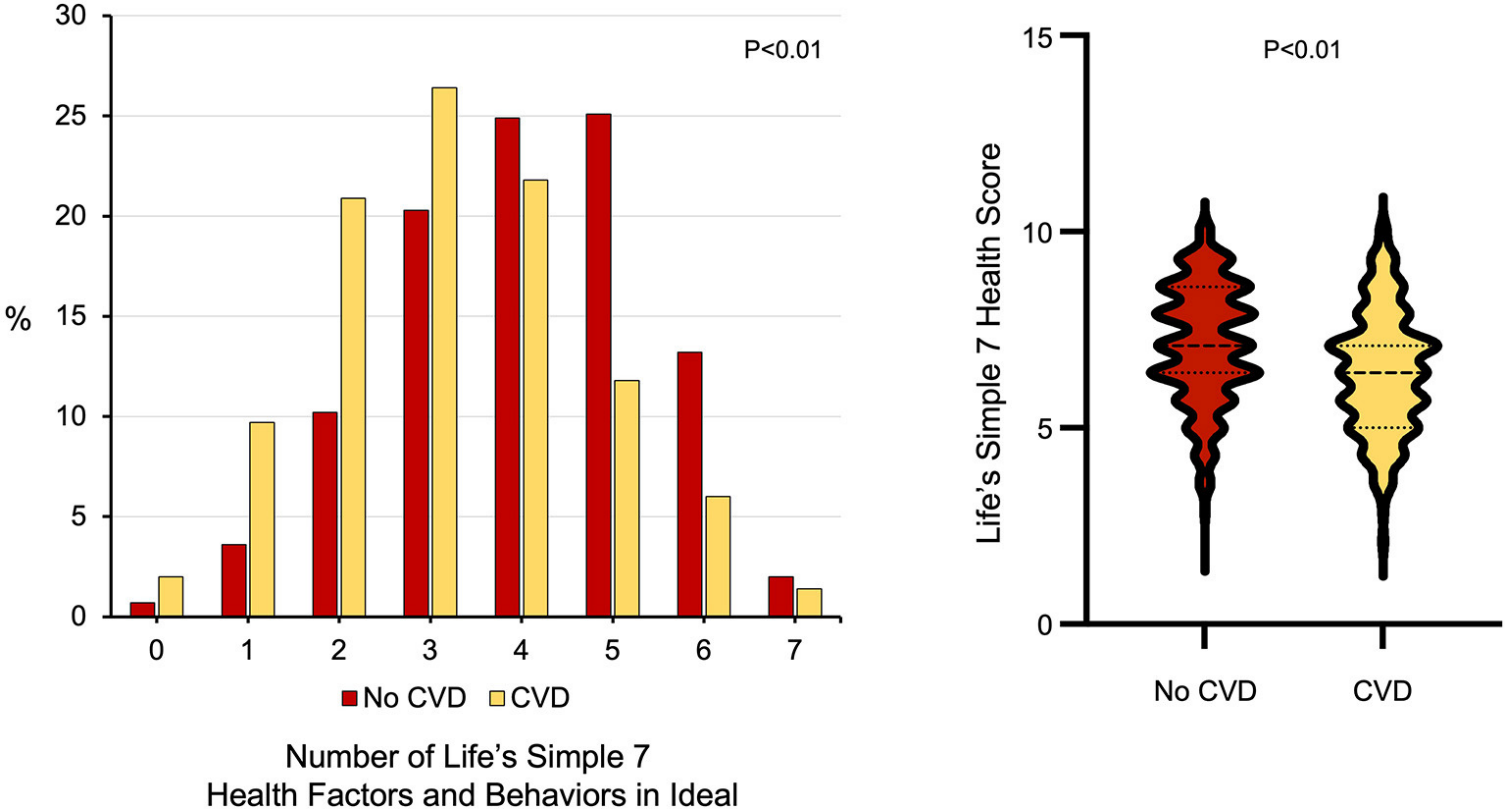
Ideal cardiovascular health in participants aged <50 years old with and without cardiovascular diseases. **(Left)** The number of Life's Simple 7 health factor and behavior categories scored as ideal for young adults with cardiovascular disease (*n* = 349) compared to those without cardiovascular disease (*n* = 696). *P* < 0.01 by Wilcoxon rank-sum test. **(Right)** Life's Simple 7 Health Score in young adults with cardiovascular disease compared to those without cardiovascular disease. *P* < 0.01 by Wilcoxon rank-sum test.

There were also differences between young adults with and without prior cardiovascular disease with respect to other modifiable cardiovascular health behaviors, including diet and dietary habits as well as weekly minutes of exercise (Table 1). Individuals with cardiovascular disease consumed fewer whole grains (*p* <0.01) and were more likely to avoid prepackaged foods, eating out, or adding salt to their food than individuals without cardiovascular disease (all *p* < 0.01). While there was no difference between the groups with respect to minutes of moderate exercise per week, young adults with cardiovascular disease self-reported fewer minutes of vigorous exercise per week than individuals without cardiovascular disease (53.8 ± 107.3 vs. 76.5 ± 118.1 min/week, *p* < 0.01). Young adults with cardiovascular disease were less likely to have ideal scores in 5 or more Life's Simple 7 health factors and behaviors categories (Figure 1) and had lower Life's Simple 7 Health Scores than young adults without cardiovascular disease (6.4 ± 1.5 vs. 7.1 ± 1.5, *p* < 0.01) (Figure 1).

Next, we examined ideal cardiovascular health in young adults with prior myocardial infarction in order to determine if our findings in young adults with the cardiovascular diseases included in our study were also observed when a single cardiovascular disease was considered. Young adults with prior myocardial infarction (*n* = 130) were compared to a matched sample of young adults without cardiovascular disease using propensity score matching that considered age, gender, race and ethnicity, and region in order minimize bias. After propensity score matching, the average effect of prior myocardial infarction on the Life's Simple 7 Health Score was a −0.8 (95% CI −1.1 to 0.4, *p* < 0.01) indicating that prior myocardial infarction was associated with a Health Score that was 0.8 points lower than that for a matched group of young adults without cardiovascular disease, similar to what was observed for the analysis that included young adults with any cardiovascular disease. A similar analysis was performed for young adults with cardiomyopathy/systolic heart failure (*n* = 107). After propensity score matching, the average effect of this cardiovascular disease was −0.7 (95% CI −1.1 to 0.4, *p* <0.01), indicating that the Health Score was decreased by 0.7 points in young adults with cardiomyopathy/systolic heart failure compared to matched young adults with no cardiovascular disease.

### Young Adults With Cardiovascular Disease Share Similarities With Older Adults With Cardiovascular Disease

**Figure 2:**
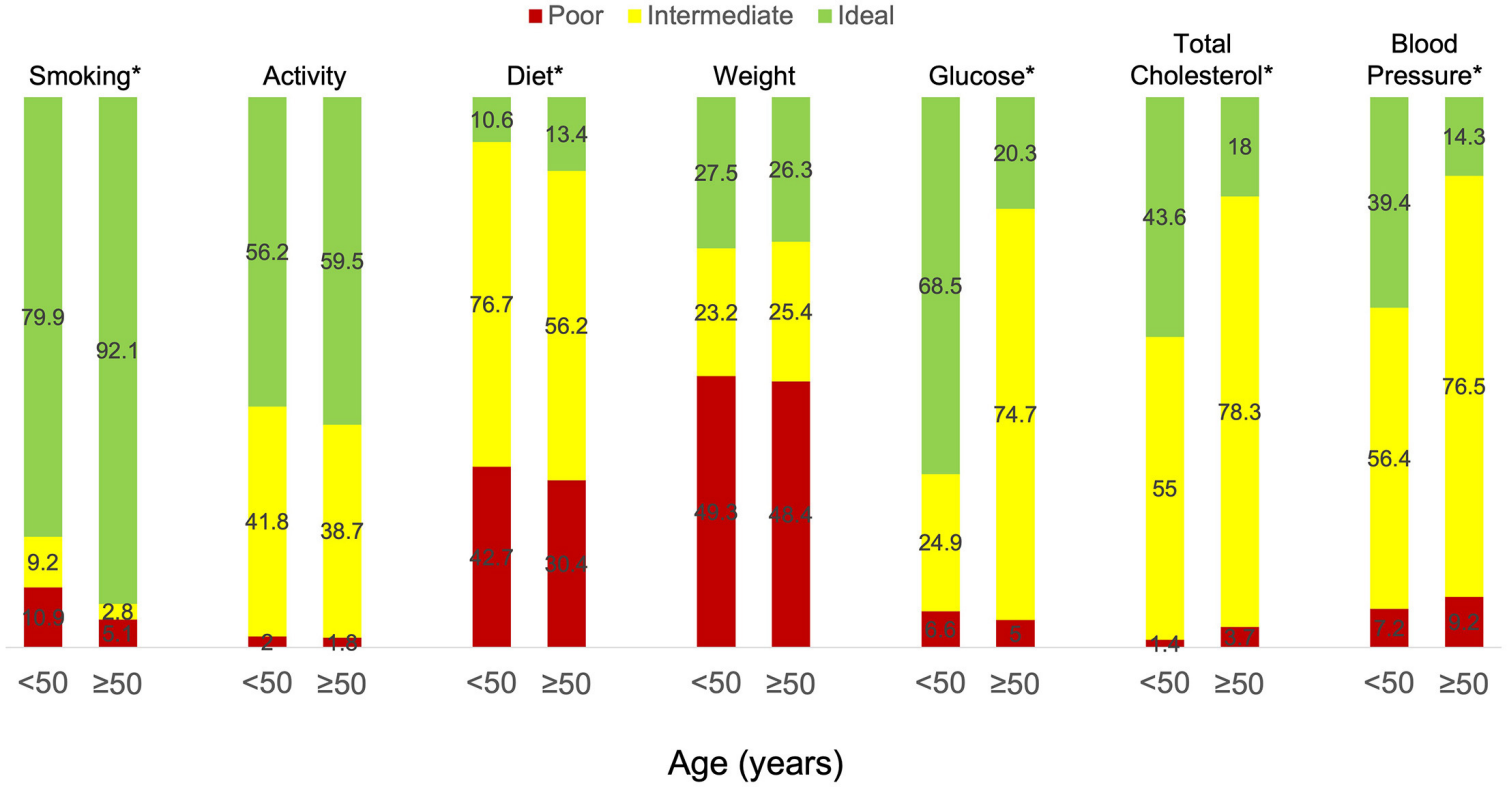
Life's Simple 7 health factors and behaviors in participants with cardiovascular disease aged <50 vs. ≥50 years old. Individuals received a score of poor, intermediate, or ideal for each of the Life's Simple 7 health factor and behaviors categories based on criteria outlined by a panel of experts. Results for young adults (age <50 years) with established cardiovascular disease (*n* = 349) are compared to those for older adults (age ≥50 years) with cardiovascular disease (*n* = 217). *p <0.01 by Wilcoxon rank-sum test.

The study also enrolled 516 individuals aged ≥50 years of whom 217 had a history of cardiovascular disease. We next sought to determine if there were similarities in ideal cardiovascular health profiles between young and older adults (aged ≥50 years) with cardiovascular disease (Table 2). In our study, young adults were more likely to be female (84.0 vs. 67.3%, *p* < 0.01) and have a lower affluence index (*p* < 0.02) than older adults, but there were no differences between the groups with respect to race or ethnicity or geographic region. Younger adults were more likely to be current or former smokers than older adults (50.4 vs. 40.1%, *p* < 0.01), had a lower prevalence of hypertension (60.2 vs. 77.4%, *p* < 0.01) and hypercholesterolemia (57.6 vs. 83.0%, *p* < 0.01) than older adults, but were less likely to be taking medications for these conditions. Young adults also had lower systolic blood pressures (116.5 ± 14.9 vs. 120.6 ± 13.8 mmHg, *p* < 0.01) and fasting blood glucose levels (99.9 ± 20.8 vs. 106.8 ± 16.3 mg/dL, *p* < 0.01) compared to older adults with no differences in diastolic blood pressure or total cholesterol levels between the groups. There were no differences between the groups with respect to other modifiable risk factors, including weight and BMI and weekly minutes of moderate or vigorous exercise. There were few differences in dietary habits between the groups with the exception of servings of fish per week, which was lower in young adults (0.9 ± 1.0 vs. 1.1 ± 1.1 servings per week, *p* < 0.01), consumption of sugar-sweetened beverages, which was higher in young adults (2.7 ± 3.4 vs. 1.5 ± 2.8, *p* < 0.01), and the tendency to avoid preprocessed foods, which was lower in young adults (55.3 vs. 64.5%, *p* < 0.04). Despite the many similarities between the groups, the percent of young adults with ideal scores in each of the Life's Simple 7 health factors and behaviors categories was lower than older adults for smoking, activity, and diet and higher than older adults for glucose, total cholesterol, and blood pressure. There were no discernable differences between the groups with respect to weight (Figure 2). Nonetheless, young adults had higher Life's Simple 7 Health Scores (6.4 ± 1.5 vs. 6.0 ± 1.4 *p* < 0.01) than older adults.

**Table 2:** Life's Simple 7 data for participants age <50 and ≥50 yrs with CVD.

	**Age <50 yrs (*n* = 349)**	**Age ≥50 yrs (*n* = 217)**	***P*-value**
Gender (no., % female)	293 (84.0)	146 (67.3)	<0.01
**Race and ethnicity (no.)**			
Asian	5	3	0.45
Black	21	7	
Hispanic	16	8	
White	290	192	
Other	17	7	
**Region (no.)**			
Northeast	59	23	0.22
South	147	97	
Midwest	75	49	
West	68	48	
Affluence status	0.39 ± 0.14	0.42 ± 0.15	<0.02
**ASCVD risk factors**			
Diabetes mellitus (no., %)	53 (15.2)	45 (20.7)	0.09
Hypertension (no., %)	210 (60.2)	168 (77.4)	<0.01
Hypercholesterolemia (no., %)	201 (57.6)	180 (83.0)	<0.01
**Medications (no., %)**			
Diabetes mellitus	46 (13.2)	39 (18.0)	0.12
Hypertension	167 (47.9)	140 (64.5)	<0.01
Hypercholesterolemia	101 (28.9)	121 (55.8)	<0.01
**Smoking status (no., %)**			
Current	38 (10.9)	11 (5.1)	<0.01
Quit ≤ 1 year	32 (9.2)	6 (2.7)	
Quit >1 year	106 (30.4)	70 (32.3)	
Never	173 (49.5)	130 (59.9)	
**Clinical data**			
Weight (kg)	87.9 ± 26.1	87.0 ± 23.2	0.69
BMI (kg/m^2^)	31.4 ± 8.7	30.7 ± 8.4	0.39
Systolic blood pressure (mmHg)*	116.5 ± 14.9	120.6 ± 13.8	<0.01
Diastolic blood pressure (mmHg)*	72.6 ± 10.2	74.1 ± 9.4	0.08
Total cholesterol (mmol/L)*	184.6 ± 29.1	183.8 ± 40.7	0.76
Blood glucose (mmol/L)*	99.9 ± 20.8	106.8 ± 16.3	<0.01
**Diet**			
Vegetables/day (cups)	1.9 ± 1.3	1.9 ± 1.2	0.70
Fruit/day (cups)	1.4 ± 1.1	1.5 ± 1.2	0.20
Fish (servings/week)	0.9 ± 1.0	1.1 ± 1.1	<0.01
Whole grains (servings/day)	1.5 ± 1.1	1.5 ± 1.1	0.90
Sugar-sweetened beverages (servings/week)	2.7 ± 3.4	1.5 ± 2.8	<0.01
Avoid prepackaged foods (no., %)	193 (55.3)	140 (64.5)	<0.04
Avoid eating out (no., %)	167 (47.9)	111 (51.2)	0.45
Avoid salt at home (no., %)	246 (70.5)	158 (72.8)	0.55
**Exercise**			
Moderate exercise (min/week)	215.5 ± 236.5	194.7 ± 198.7	0.28
Vigorous exercise (min/week)	53.8 ± 107.3	60.6 ± 119.8	0.48
**LS7 Health Score**	6.4 ± 1.5	6.0 ± 1.4	<0.01

*Contains imputed data from Life's Simple 7. Categorical variables are analyzed by Chi-Square test. Continuous variables are analyzed by t-test. Non-parametric variables were analyzed by Wilcoxon rank-sum test. CVD, cardiovascular disease; ASCVD, atherosclerotic cardiovascular disease.

### Heterogeneity Among Young Adults With Cardiovascular Disease

We next sought to explore the group of young adults with cardiovascular disease in more detail to determine if there were subgroups of individuals with differences in ideal cardiovascular health habits and behaviors. To identify these subgroups, we performed a latent class analysis. In our population of young adults with prior cardiovascular disease, analysis of latent class models indicated that the best fit was provided by a two-class model with a Vuong-Lo-Mendell-Rubin likelihood ratio test *p* < 0.03. In this model, the average probability of for class 1 was 0.87 and for class 2 was 0.82 indicating good model fit. This resulted in 173 individuals assigned to phenogroup 1 and 176 individuals assigned to phenogroup 2. Participants assigned to phenogroup 1 were younger (38.2 ± 7.1 vs. 41.4 ± 6.0 yrs, *p* < 0.01) than participants assigned to phenogroup 2 with no differences in gender, race or ethnicity, or geographic location between the groups. Participants in phenogroup 1 had a substantially lower prevalence of diabetes mellitus (1.7 vs. 28.4%, *p* < 0.01), hypertension (33.0 vs. 86.9%, *p* < 0.01), and hypercholesterolemia (45.1 vs. 69.6%, *p* < 0.01) as well as medication use for these risk factors (all *p* < 0.01) than participants in phenogroup 2. Phenogroup 1 had lower systolic (110.8 ± 11.4 vs. 122.0 ± 15.8 mmHg, *p* <0.01) and diastolic (68.5 ± 7.3 vs. 76.6 ± 11.1 mmHg, *p* < 0.01) blood pressures and fasting blood glucose levels (93.4 ± 7.5 vs. 106.2 ± 26.9 mg/dL, *p* < 0.01) with similar cholesterol levels as phenogroup 2 individuals. There were no differences between the phenogroups with respect to consumption of a heart healthy diet and following heart healthy dietary habits. Although participants in both phenogroups performed the same amount of weekly moderate exercise (233.1 ± 219.3 vs. 208.0 ± 252.7 min/week, *p* = 0.55), individuals in phenogroup 1 performed more weekly vigorous exercise (76.4 ± 126.9 vs. 31.5 ± 77.9 min/week, *p* < 0.01) than those in phenogroup 2. As a result, participants in phenogroup 1 also had significantly higher Life's Simple 7 Health Scores compared to phenogroup 2 (7.4 ± 1.2 vs. 5.5 ± 1.1, *p* < 0.01).

### Digital Health Device Data

A subgroup of young adults contributed weight (*n* = 210), activity (*n* = 247), and step count (*n* = 262) data from digital health devices. Weight measured by digital health devices and the corresponding calculated body mass index remained numerically higher in young adults with cardiovascular disease (*n* = 77) as compared to individuals without cardiovascular disease (*n* = 133) (Table 3). When comparing self-reported with digital health device measured weight, on average weight was underreported by both young adults with cardiovascular disease (−0.9 ± 5.7 kg) and without cardiovascular disease (−0.3 ± 3.5 kg). Using digital health device measured weight, differences in the distribution of participants with poor, intermediate, and ideal scores persisted with a greater percentage of young adults with prior cardiovascular disease having poor weight scores (*p* < 0.05). Using digital health device measured exercise data, participants with prior cardiovascular disease performed a similar number of minutes per week of moderate and vigorous exercise as individuals without cardiovascular disease. When comparing self-reported with digital health device measured activity, young adults with cardiovascular disease on average overreported weekly minutes of moderate exercise (67.5 ± 226.0 min) and underreported weekly minutes of vigorous exercise (−79.3 ± 188.0 min). A similar pattern was seen for young adults without cardiovascular disease who overreported weekly minutes of moderate exercise (85.2 ± 274.6 min) and underreported weekly minutes of vigorous exercise (−34.6 ± 177.2 min). Using digital health device measured exercise data resulted in a similar distribution of participants with intermediate and ideal activity scores between the groups. Participants in both groups also recorded a similar number of daily steps (Table 3), suggesting a similar amount of contact time with their digital health devices.

**Table 3:** Digital health device measured weight and exercise data for participants age <50 yrs with or without CVD.

	**Age <50 yrs No CVD**	**Age <50 yrs CVD**	***P*-value**
	**(*****n*** **=** **133)**	**(*****n*** **=** **77)**	
Weight (kg)	80.3 ± 20.4	82.6 ± 23.9	0.45
Delta reported vs. measured weight (kg)	−0.3 ± 3.5	−0.9 ± 5.7	0.35
BMI (kg/m^2^)	28.2 ± 6.7	29.6 ± 8.1	0.17
Delta reported vs. measured BMI (kg/m^2^)	−0.1 ± 1.3	−0.4 ± 2.0	0.37
**Healthy weight score (no., %)**			
Poor	40 (30.1)	35 (45.4)	<0.05
Intermediate	42 (31.6)	19 (24.7)	
Ideal	51 (38.3)	23 (29.9)	
	**(*****n*** **=** **170)**	**(*****n*** **=** **77)**	
Moderate exercise (min/week)	127.6 ± 161.3	117.7 ± 143.3	0.64
Delta reported vs. measured moderate exercise (min/week)	85.2 ± 274.6	67.5 ± 226.0	0.62
Vigorous exercise (min/week)	136.5 ± 176.1	137.3 ± 181.6	0.97
Delta reported vs. measured vigorous exercise (min/week)	−34.6 ± 177.2	−79.3 ± 188.0	0.07
**Physical Activity Score (no., %)**			
Poor	0 (0.0)	0 (0.0)	0.82
Intermediate	68 (40.0)	32 (41.6)	
Ideal	102 (60.0)	45 (58.4)	
	**(*****n*** **=** **123)**	**(*****n*** **=** **72)**	
Recalculated LS7 Health Score	7.4 ± 1.2	6.9 ± 1.3	<0.03
	**(*****n*** **=** **179)**	**(*****n*** **=** **83)**	
Steps per day	7,402.4 ± 4,553.2	7,537.7 ± 3,960.4	0.82

*Categorical variables are analyzed by Chi-Square test. Continuous variables are analyzed by t-test. Non-parametric variables were analyzed by Wilcoxon rank-sum test*.

## Discussion

In this analysis of ideal cardiovascular health in young adults with cardiovascular disease, we found such individuals were less likely to achieve ideal scores in 5 or more Life's Simple 7 cardiovascular health and behavior categories and had lower Health Scores compared to young adults without cardiovascular disease. We identified similarities in ideal cardiovascular health categories between young and older adults with cardiovascular disease, including similar body mass index, dietary habits, and exercise patterns. In a subset of young adults, we also examined the effect of substituting digital health device data for self-reported data to assess ideal cardiovascular health. Here, we found that substituting digital health device activity data for self-reported data resulted in improvements in the activity category score for young adults with cardiovascular disease. When comparing self-reported and digital health device measured weight and exercise activity data, we noted that individuals tended to underreport their weight and weekly minutes of vigorous activity while overreporting their weekly minutes of moderate activity. The observed difference between self-reported and measured data is in line with what was observed in the entire My Research Legacy study sample (21).

We also explored heterogeneity among young adults enrolled in the study using latent class analysis and found that there were two phenogroups. These phenogroups were significantly different for 6 of the 7 ideal cardiovascular health and behavior categories. Compared to phenogroup 1, young adults with cardiovascular disease assigned to phenogroup 2 had a significantly higher body mass index, prevalence of cardiovascular disease risk factors, and performed fewer minutes of weekly vigorous exercise, which is consistent with their lower overall Health Scores. The ramifications of a lower Health Score have been determined in a longitudinal study that reported that each one-point increase in the Health Score was associated with a 12% decreased risk of major adverse cardiovascular events when the Life's Simple 7 Health Score was calculated on a 0–14-point scale (31). The heterogeneity identified between these phenogroups also highlights the substantial differences in the health factors and behaviors that comprise ideal cardiovascular health among young adults with established disease. This finding suggests that members of phenogroup 1 may have made optimized their cardiovascular health factors and behaviors following their event or they may have had an event despite having ideal cardiovascular health. This latter explanation is plausible as the event may be attributable to mechanisms other than typical atherosclerotic cardiovascular disease associated with cardiovascular events in older individuals, such as plaque erosion, microvascular disease, vasospasm, or spontaneous coronary artery dissection (32). In contrast, members of phenogroup 2 with less-than-ideal cardiovascular health represent a group of young adults that has yet to optimize their cardiovascular health factors and behaviors and may be at higher risk for recurrent events. This hypothesis is supported by studies that have demonstrated increased risk of cardiovascular events in young adults with hypertension (33), hypercholesterolemia (34), diabetes mellitus (35), obesity (36), combustible tobacco use (37), unhealthy diet (38), and sedentary behavior (39, 40).

The importance of assessing ideal cardiovascular health in young adults is necessary as ideal levels of cardiovascular health factors and behaviors decline with age. In NHANES, the prevalence of young adults aged 20–39 years with ideal blood pressure, blood glucose, total cholesterol, weight, and diet who abstain from use of combustible tobacco was lower than in teenagers aged 12–19 years. Although the percentage of young adults with ideal physical activity was higher compared to teenagers, it was also higher than in adults aged 40–59 years, suggesting that ideal physical activity also declines with aging (1).

Assessing modifiable cardiovascular health factors and habits in young adults also has important ramifications for cardiovascular risk prediction. This was demonstrated in a study of 36,030 individuals enrolled in 6 community-based cohort studies. When exposures as a young adult were considered, elevated levels of low-density lipoprotein cholesterol and diastolic blood pressure were associated with a 64% increased risk of coronary heart disease while elevated systolic blood pressure was associated with a 37% increased risk of heart failure (41). Similarly, physical inactivity assessed as cumulative sedentary behaviors was also associated with an increased risk of cardiovascular disease (pooled relative risk, 2.47; 95% CI, 1.44–4.24) (42, 43). Exploratory studies have also linked physical inactivity to a decrease in insulin sensitivity and diminished conduit and resistance artery compliance compared to what was observed in physically active young adults (44).

Ideal cardiovascular health in young adults is affected by several factors, including awareness, behaviors, as well as socioeconomic status. Socioeconomic status has been shown to influence cardiovascular risk factors and this relationship holds regardless of the socioeconomic risk indicator (e.g., income, occupation, education) studied or if analyzed at the individual, family, or neighborhood level (45). Our study found that young adults with cardiovascular disease had a lower affluence index than those without, which is consistent with prior reports. Ideal cardiovascular health may also be related to awareness of cardiovascular risk factors, which was analyzed in 11,083 young adults aged 18–39 years in the United States. Awareness of hypercholesterolemia, hypertension, and diabetes mellitus exceeded 60%; however, awareness of borderline increased levels of blood glucose, cholesterol, and blood pressure was significantly lower (5.7, 22.5, and 12.3%, respectively). Levels of awareness were affected by race and ethnicity, educational level, and socioeconomic status with lower levels reported in Blacks and Hispanics as well as individuals with lower socioeconomic status (46).

In our study, the definition of cardiovascular disease included myocardial infarction, stroke, cardiomyopathy/systolic heart failure, atrial fibrillation, and aortic dissection in order to capture a broad representation of cardiovascular diseases in young adults. To date, there are few studies that have focused solely on cardiovascular health and disease in young adults, and none explored ideal cardiovascular health in young adults with established cardiovascular diseases. Two large community-based studies, the Coronary Artery Risk Development in Young Adults (CARDIA) study and the Young Finns Study, examined the relationship between risk factors and incident atherosclerotic cardiovascular disease or subclinical atherosclerosis, respectively (20). The CARDIA study enrolled 5,115 young adults aged 18–30 years and confirmed that traditional risk factors assessed in the Life's Simple 7 survey, including age, sex, hypertension, diabetes mellitus, and hypercholesterolemia, correlated with coronary calcium levels (47). The Young Finns Study, which enrolled 3,596 individuals aged 3–18, reported that obesity, tobacco use, hypertension, and hyperlipidemia predicted subclinical atherosclerosis in young adults (48). Additional analyses also associated diet and physical activity, both assessed as part of the Life's Simple 7 survey, with subclinical atherosclerosis (49). In contrast to our study, young adults with cardiovascular disease were not enrolled and ideal cardiovascular health wasn't assessed.

Several large community-based studies that included young adults analyzed the relationship between ideal cardiovascular health (assessed by the Life's Simple 7 survey tool) and incident cardiovascular diseases. The Framingham Heart Study, the Multi-Ethnic Study of Atherosclerosis (MESA), and the Atherosclerosis Risk in Communities (ARIC) studies have all reported that ideal cardiovascular health was associated with a lower risk of incident atherosclerotic cardiovascular disease (16, 50–52), heart failure (15, 53), and atrial fibrillation (54). However, none of these studies examined ideal cardiovascular health specifically in young adults or in young adults with established cardiovascular disease. Therefore, our study design, which included participants with and without established cardiovascular disease, has implications for future studies that examine ideal cardiovascular health in real world samples of young adults. These studies should include individuals with established cardiovascular disease and the definition of cardiovascular diseases should be sufficiently broad to ensure representation of typical cardiovascular diseases prevalent in young adults. Future studies should also be broadly inclusive in order to examine the role of gender, race, and ethnicity, as well as social determinants of health on ideal cardiovascular health in young adults.

Our study was enriched for young women with cardiovascular disease (female to male ratio of 4.6:1), which is not surprising as recent analyses from community-based studies have identified an increase in cardiovascular disease in young women. An analysis from ARIC of 20-year trends for myocardial infarction in young adults (age 35–54 years) reported that the largest increase in the annual incidence of hospitalization for myocardial infarction was seen in young women. Compared to young men with myocardial infarction, young women had a higher burden of comorbidities, were more often Black, and were less likely to be treated with guideline-directed medical or invasive therapies (3). A similar phenomenon has also been observed for young women with stroke. A retrospective analysis of an insurance claims database in the United States demonstrated that the incidence rate ratio of men to women was 0.70 (95% CI, 0.57–0.86) for individuals aged 25–34 years and 0.87 (95% CI, 0.78–0.98), for individuals aged 35–44 years, indicating that more young women than young men had strokes (55). Similarly, the European 15 Cities Young Stroke Study reported that there were 30% more strokes in young women than young men (56).

Our study has several limitations that have the potential to affect the generalizability of our findings. First, our study enrolled a relatively small sample size compared to larger community-based studies, such as NHANES. We also considered several cardiovascular diseases, including myocardial infarction, stroke, atrial fibrillation, systolic heart failure/cardiomyopathy, and aortic dissection, as a composite of prevalent cardiovascular diseases in young adults (20). Since our study design was direct-to-participant, we relied on participant responses to disease-specific questions written by a panel of experts to establish the diagnosis of cardiovascular disease and were not able to review electronic health or other medical records. Several studies assessed the accuracy of self-reported cardiovascular diseases and reported that the positive predictive value for myocardial infarction ranged from 39 to 84%, and 33 to 81% for stroke. These studies, however, were conducted in older patient populations and it is likely that there are notable differences between the participants enrolled in these studies and our young adult population (57, 58). Our study did not collect a family history of cardiovascular disease and it is not assessed as part of the Life's Simple 7 survey. Family history, which is considered an indicator of shared genetic and environmental exposures, is a well-recognized risk factor for cardiovascular diseases (59). A family history of cardiovascular disease as well as a family history of premature cardiovascular disease have been shown to associate independently with coronary heart disease risk and incident atherosclerotic cardiovascular disease and, therefore, are important determinants of additional risk information for asymptomatic individuals (60). By study design, we examined digital health device data in only a subset of participants and, therefore, were underpowered to analyze the effect of substituting digital health data for self-reported data for individual cardiovascular diseases. Finally, we do not have longitudinal outcome data to determine if there are shifts between the phenogroups of young adults with cardiovascular disease over time.

In summary, our study demonstrated that young adults with established cardiovascular diseases have less ideal cardiovascular health factors and habits than young adults without disease and more closely resemble older adults with cardiovascular disease. Our sample of young adults with cardiovascular disease was heterogeneous and comprised of two phenogroups with different cardiovascular risk profiles indicating that ideal cardiovascular health in young adults with established disease is complex. The relationship between these phenogroups and clinical outcomes warrants investigation in future studies as our findings may have implications for designing behavioral and therapeutic interventions in the future to improve cardiovascular morbidity and mortality.

## Data Availability Statement

The data are not publicly available due to privacy restrictions and protection of personal data. Requests to access the datasets should be directed to Jane Leopold, jleopold@bwh.harvard.edu.

## Ethics Statement

The studies involving human participants were reviewed and approved by Advarra Institutional Review Board. The patients/participants provided their written informed consent to participate in this study.

## Author Contributions

JL and EA: conceptualization, investigation, writing—original draft preparation, and writing—review and editing. JL: formal analysis and data curation. Both authors have read and agreed to the published version of the manuscript.

## Funding

This research was funded by the American Heart Association AIM 19AIML34980000, the National Institutes of Health, and National Heart, Lung, and Blood Institute U01 HL125215 (JL).

## Conflict of Interest

The authors declare that the research was conducted in the absence of any commercial or financial relationships that could be construed as a potential conflict of interest.

## Publisher's Note

All claims expressed in this article are solely those of the authors and do not necessarily represent those of their affiliated organizations, or those of the publisher, the editors and the reviewers. Any product that may be evaluated in this article, or claim that may be made by its manufacturer, is not guaranteed or endorsed by the publisher.

## References

[1] Gooding HC Gidding SS Moran AE Redmond N Allen NB Bacha F. Challenges and opportunities for the prevention and treatment of cardiovascular disease among young adults: report from a national heart, lung, and blood institute working group. J Am Heart Assoc. (2020) 9:e016115. 10.1161/JAHA.120.016115 32993438 PMC7792379

[2] O'Flaherty M Buchan I Capewell S. Contributions of treatment and lifestyle to declining CVD mortality: why have CVD mortality rates declined so much since the 1960s? Heart. (2013) 99:159–62. 10.1136/heartjnl-2012-302300 22962283

[3] Arora S Stouffer GA Kucharska-Newton AM Qamar A Vaduganathan M Pandey A. Twenty year trends and sex differences in young adults hospitalized with acute myocardial infarction. Circulation. (2019) 139:1047–56. 10.1161/CIRCULATIONAHA.118.037137 30586725 PMC6380926

[4] Ford ES Capewell S. Coronary heart disease mortality among young adults in the U.S. from 1980 through 2002: concealed leveling of mortality rates. J Am Coll Cardiol. (2007) 50:2128–32. 10.1016/j.jacc.2007.05.056 18036449

[5] Ford ES. Trends in predicted 10-year risk of coronary heart disease and cardiovascular disease among U.S. adults from 1999 to 2010. J Am Coll Cardiol. (2013) 61:2249–52. 10.1016/j.jacc.2013.03.023 23563124 PMC4571185

[6] George MG Tong X Bowman BA. Prevalence of cardiovascular risk factors and strokes in younger adults. JAMA Neurol. (2017) 74:695–703. 10.1001/jamaneurol.2017.0020 28395017 PMC5559660

[7] Pearson-Stuttard J Guzman-Castillo M Penalvo JL Rehm CD Afshin A Danaei G. Modeling future cardiovascular disease mortality in the United States: national trends and racial and ethnic disparities. Circulation. (2016) 133:967–78. 10.1161/CIRCULATIONAHA.115.019904 26846769 PMC4783256

[8] Leppert MH Poisson SN Sillau SH Campbell JD Ho PM Burke JF. Is prevalence of atherosclerotic risk factors increasing among young adults? It depends on how you ask. J Am Heart Assoc. (2019) 8:e010883. 10.1161/JAHA.118.010883 30857455 PMC6475043

[9] Brown AF Liang LJ Vassar SD Escarce JJ Merkin SS Cheng E. Trends in racial/ethnic and nativity disparities in cardiovascular health among adults without prevalent cardiovascular disease in the United States, 1988 to 2014. Ann Intern Med. (2018) 168:541–9. 10.7326/M17-0996 29554692 PMC6499476

[10] Egan BM Li J Sutherland SE Jones DW Ferdinand KC Hong Y. Sociodemographic determinants of life's simple 7: implications for achieving cardiovascular health and health equity goals. Ethn Dis. (2020) 30:637–650. 10.18865/ed.30.4.637 32989364 PMC7518524

[11] Lloyd-Jones DM Hong Y Labarthe D Mozaffarian D Appel LJ Van Horn L. Defining and setting national goals for cardiovascular health promotion and disease reduction: the American heart association's strategic impact goal through 2020 and beyond. Circulation. (2010) 121:586–613. 10.1161/CIRCULATIONAHA.109.192703 20089546

[12] Angell SY McConnell MV Anderson CAM Bibbins-Domingo K Boyle DS Capewell S. The American heart association 2030 impact goal: a presidential advisory from the American heart association. Circulation. (2020) 141:e120–38. 10.1161/CIR.0000000000000758 31992057 PMC8690536

[13] Sanchez E. Life's simple 7: vital but not easy. J Am Heart Assoc. (2018) 7:1–4. 10.1161/JAHA.118.009324 29773574 PMC6015360

[14] Collins TC Slovut DP Newton Jr R Johnson WD Larrivee S. Ideal cardiovascular health and peripheral artery disease in African Americans: results from the jackson heart study. Prev Med Rep. (2017) 7:20–5. 10.1016/j.pmedr.2017.05.005 28593118 PMC5447374

[15] Nayor M Enserro DM Vasan RS Xanthakis V. Cardiovascular health status and incidence of heart failure in the framingham offspring study. Circ Heart Fail. (2016) 9:e002416. 10.1161/CIRCHEARTFAILURE.115.002416 26699391 PMC4692176

[16] Enserro DM Vasan RS Xanthakis V. Twenty-year trends in the American heart association cardiovascular health score and impact on subclinical and clinical cardiovascular disease: the framingham offspring study. J Am Heart Assoc. (2018) 7:1–9. 10.1161/JAHA.118.008741 29773573 PMC6015351

[17] Spahillari A Talegawkar S Correa A Carr JJ Terry JG Lima J. Ideal cardiovascular health, cardiovascular remodeling, and heart failure in blacks: the jackson heart study. Circ Heart Fail. (2017) 10:1–9. 10.1161/CIRCHEARTFAILURE.116.003682 28209767 PMC5319800

[18] Corlin L Short MI Vasan RS Xanthakis V. Association of the duration of ideal cardiovascular health through adulthood with cardiometabolic outcomes and mortality in the framingham offspring study. JAMA Cardiol. (2020) 5:549–56. 10.1001/jamacardio.2020.0109 32159731 PMC7066529

[19] Bundy JD Zhu Z Ning H Zhong VW Paluch AE Wilkins JT. Estimated impact of achieving optimal cardiovascular health among US adults on cardiovascular disease events. J Am Heart Assoc. (2021) 10:e019681. 10.1161/JAHA.120.019681 33761755 PMC8174373

[20] Andersson C Vasan RS. Epidemiology of cardiovascular disease in young individuals. Nat Rev Cardiol. (2018) 15:230–40. 10.1038/nrcardio.2017.154 29022571

[21] Leopold JA Davis RB Antman EM. Data from digital health devices informs ideal. Cardiovasc Health. (2021) 11:189–201. 10.3390/jpm11030189 33801949 PMC7998383

[22] Clarke P Morenoff J Debbink M Golberstein E Elliott MR Lantz PM. Cumulative exposure to neighborhood context: consequences for health transitions over the adult life course. Res Aging. (2014) 36:115–42. 10.1177/0164027512470702 24465068 PMC3900407

[23] Clarke PJ, Melendez, R,. National Neighborhood Data Archive (NaNDA): Neighborhood Socioeconomic Demographic Characteristics of Census Tracts, United States, 2000-2010 (V1) [Data set]. Inter-university Consortium for Political Social Research. (2019). Available online at: www.openicpsr.org (accessed May 17, 2021).

[24] Virani SS Alonso A Benjamin EJ Bittencourt MS Callaway CW Carson AP. Heart disease and stroke statistics-2020 update: a report from the American heart association. Circulation. (2020) 141:e139–596. 10.1161/CIR.0000000000000757 31992061

[25] Haghayegh S Khoshnevis S Smolensky MH Diller KR Castriotta RJ. Accuracy of wristband fitbit models in assessing sleep: systematic review and meta-analysis. J Med Internet Res. (2019) 21:e16273. 10.2196/16273 31778122 PMC6908975

[26] Sinha P Calfee CS Delucchi KL. Practitioner's guide to latent class analysis: methodological considerations and common pitfalls. Crit Care Med. (2021) 49:e63–79. 10.1097/CCM.0000000000004710 33165028 PMC7746621

[27] Ahanchi NS Hadaegh F Azizi F Khalili D. Sex- specific clustering of metabolic syndrome components and incidence of cardiovascular disease: a latent class analysis in a population-based cohort study. J Diabetes Compl. (2021) 35:107942. 10.1016/j.jdiacomp.2021.107942 33965337

[28] Kongsted A Nielsen AM. Latent class analysis in health research. J Physiother. (2017) 63:55–58. 10.1016/j.jphys.2016.05.018 27914733

[29] Feuillet F Bellanger L Hardouin JB Victorri-Vigneau C Sebille V. On comparison of clustering methods for pharmacoepidemiological data. J Biopharm Stat. (2015) 25:843–56. 10.1080/10543406.2014.920855 24905478

[30] Hagenaars JA McCutcheon AL. Applied Latent Class Analysis. 1st ed. New York, NY: Cambridge University Press (2002). 480 p.

[31] Nguyen ATH Saeed A Bambs CE Swanson J Emechebe N Mansuri F. Usefulness of the American heart association's ideal cardiovascular health measure to predict long-term major adverse cardiovascular events (From the Heart SCORE Study). Am J Cardiol. (2021) 138:20–5. 10.1016/j.amjcard.2020.10.019 33065086

[32] Dreyer RP Dharmarajan K Kennedy KF Jones PG Vaccarino V Murugiah K. Sex differences in 1-Year all-cause rehospitalization in patients after acute myocardial infarction: a prospective observational study. Circulation. (2017) 135:521–31. 10.1161/CIRCULATIONAHA.116.024993 28153989 PMC5312975

[33] Singh A Collins BL Gupta A Fatima A Qamar A Biery D. Cardiovascular risk and statin eligibility of young adults after an MI: partners YOUNG-MI registry. J Am Coll Cardiol. (2018) 71:292–302. 10.1016/j.jacc.2017.11.007 29141201 PMC5831171

[34] Jeong SM Choi S Kim K Kim SM Lee G Park SY. Effect of change in total cholesterol levels on cardiovascular disease among young adults. J Am Heart Assoc. (2018) 7:1–9. 10.1161/JAHA.118.008819 29899019 PMC6220545

[35] Rawshani A Sattar N Franzen S Rawshani A Hattersley AT Svensson AM. Excess mortality and cardiovascular disease in young adults with type 1 diabetes in relation to age at onset: a nationwide, register-based cohort study. Lancet. (2018) 392:477–86. 10.1016/S0140-6736(18)31506-X 30129464 PMC6828554

[36] Khan SS Ning H Wilkins JT Allen N Carnethon M Berry JD. Association of body mass index with lifetime risk of cardiovascular disease and compression of morbidity. JAMA Cardiol. (2018) 3:280–7. 10.1001/jamacardio.2018.0022 29490333 PMC5875319

[37] Khan SS Ning H Sinha A Wilkins J Allen NB Vu THT. Cigarette smoking and competing risks for fatal and nonfatal cardiovascular disease subtypes across the life course. J Am Heart Assoc. (2021) 10:e021751. 10.1161/JAHA.121.021751 34787470 PMC9075374

[38] Gooding HC Ning H Gillman MW Shay C Allen N Goff DC. Application of a lifestyle-based tool to estimate premature cardiovascular disease events in young adults: the coronary artery risk development in young adults (CARDIA) study. JAMA Intern Med. (2017) 177:1354–60. 10.1001/jamainternmed.2017.2922 28715555 PMC5710563

[39] Garcia JM Duran AT Schwartz JE Booth JN 3rd Hooker SP Willey JZ. Types of sedentary behavior and risk of cardiovascular events and mortality in blacks: the jackson heart study. J Am Heart Assoc. (2019) 8:e010406. 10.1161/JAHA.118.010406 31238767 PMC6662345

[40] Pandey A Salahuddin U Garg S Ayers C Kulinski J Anand V. Continuous dose-response association between sedentary time and risk for cardiovascular disease: a meta-analysis. JAMA Cardiol. (2016) 1:575–83. 10.1001/jamacardio.2016.1567 27434872

[41] Zhang Y Vittinghoff E Pletcher MJ Allen NB Zeki Al Hazzouri A Yaffe K. Associations of blood pressure and cholesterol levels during young adulthood with later cardiovascular events. J Am Coll Cardiol. (2019) 74:330–41. 10.1016/j.jacc.2019.03.529 31319915 PMC6764095

[42] Wilmot EG Edwardson CL Biddle SJ Gorely T Henson J Khunti K. Prevalence of diabetes and impaired glucose metabolism in younger 'at risk' UK adults: insights from the STAND programme of research. Diabet Med. (2013) 30:671–5. 10.1111/dme.12173 23506383

[43] Young DR Hivert MF Alhassan S Camhi SM Ferguson JF Katzmarzyk PT. Sedentary behavior and cardiovascular morbidity and mortality: a science advisory from the American heart association. Circulation. (2016) 134:e262–79. 10.1161/CIR.0000000000000440 27528691

[44] McGavock JM Anderson TJ Lewanczuk RZ. Sedentary lifestyle and antecedents of cardiovascular disease in young adults. Am J Hypertens. (2006) 19:701–7. 10.1016/j.amjhyper.2006.01.013 16814124

[45] Adler NE Stewart J. Health disparities across the lifespan: meaning, methods, and mechanisms. Ann N Y Acad Sci. (2010) 1186:5–23. 10.1111/j.1749-6632.2009.05337.x 20201865

[46] Bucholz EM Gooding HC de Ferranti SD. Awareness of cardiovascular risk factors in U.S. Young adults aged 18-39 years. Am J Prev Med. (2018) 54:e67–77. 10.1016/j.amepre.2018.01.022 29433955 PMC5893222

[47] Carr JJ Jacobs Jr DR Terry JG Shay CM Sidney S Liu K. Association of coronary artery calcium in adults aged 32 to 46 years with incident coronary heart disease and death. JAMA Cardiol. (2017) 2:391–9. 10.1001/jamacardio.2016.5493 28196265 PMC5397328

[48] Hartiala O Magnussen CG Kajander S Knuuti J Ukkonen H Saraste A. Adolescence risk factors are predictive of coronary artery calcification at middle age: the cardiovascular risk in young finns study. J Am Coll Cardiol. (2012) 60:1364–70. 10.1016/j.jacc.2012.05.045 22981553

[49] Juonala M Viikari JS Raitakari OT. Main findings from the prospective cardiovascular risk in young finns study. Curr Opin Lipidol. (2013) 24:57–64. 10.1097/MOL.0b013e32835a7ed4 23069987

[50] Gaye B Tajeu GS Vasan RS Lassale C Allen NB Singh-Manoux A. Association of changes in cardiovascular health metrics and risk of subsequent cardiovascular disease and mortality. J Am Heart Assoc. (2020) 9:e017458. 10.1161/JAHA.120.017458 32985301 PMC7792367

[51] Folsom AR Yatsuya H Nettleton JA Lutsey PL Cushman M Rosamond WD. Community prevalence of ideal cardiovascular health, by the American heart association definition, and relationship with cardiovascular disease incidence. J Am Coll Cardiol. (2011) 57:1690–6. 10.1016/j.jacc.2010.11.041 21492767 PMC3093047

[52] Unkart JT Allison MA Criqui MH McDermott MM Wood AC Folsom AR. Life's simple 7 and peripheral artery disease: the multi-ethnic study of atherosclerosis. Am J Prev Med. (2019) 56:262–70. 10.1016/j.amepre.2018.09.021 30553692 PMC6422346

[53] Ogunmoroti O Oni E Michos ED Spatz ES Allen NB Rana JS. Life's simple 7 and incident heart failure: the multi-ethnic study of atherosclerosis. J Am Heart Assoc. (2017) 6:1–9. 10.1161/JAHA.116.005180 28655734 PMC5669160

[54] Ogunmoroti O Michos ED Aronis KN Salami JA Blankstein R Virani SS. Life's simple 7 and the risk of atrial fibrillation: the multi-ethnic study of atherosclerosis. Atherosclerosis. (2018) 275:174–81. 10.1016/j.atherosclerosis.2018.05.050 29920438

[55] Leppert MH Ho PM Burke J Madsen TE Kleindorfer D Sillau S. Young women had more strokes than young men in a large, United States claims sample. Stroke. (2020) 51:3352–5. 10.1161/STROKEAHA.120.030803 32942966 PMC7606353

[56] Putaala J Yesilot N Waje-Andreassen U Pitkaniemi J Vassilopoulou S Nardi K. Demographic and geographic vascular risk factor differences in European young adults with ischemic stroke: the 15 cities young stroke study. Stroke. (2012) 43:2624–30. 10.1161/STROKEAHA.112.662866 22798330

[57] Bergmann MM Byers T Freedman DS Mokdad A. Validity of self-reported diagnoses leading to hospitalization: a comparison of self-reports with hospital records in a prospective study of American adults. Am J Epidemiol. (1998) 147:969–77. 10.1093/oxfordjournals.aje.a009387 9596475

[58] Heckbert SR Kooperberg C Safford MM Psaty BM Hsia J McTiernan A. Comparison of self-report, hospital discharge codes, and adjudication of cardiovascular events in the Women's health initiative. Am J Epidemiol. (2004) 160:1152–8. 10.1093/aje/kwh314 15583367

[59] Lloyd-Jones DM Nam BH D'Agostino RB Sr., Levy D, Murabito JM, et al. Parental cardiovascular disease as a risk factor for cardiovascular disease in middle-aged adults: a prospective study of parents and offspring. JAMA. (2004) 291:2204–11. 10.1001/jama.291.18.2204 15138242

[60] Mehta A Virani SS Ayers CR Sun W Hoogeveen RC Rohatgi A. Lipoprotein(a) and family history predict cardiovascular disease risk. J Am Coll Cardiol. (2020) 76:781–93. 10.1016/j.jacc.2020.06.040 32792075

