# Supplementary material for: Ideal Cardiovascular Health in Young Adults With Established Cardiovascular Diseases
Source: Front Cardiovasc Med. 2022 Feb 17;9:814610. doi: 10.3389/fcvm.2022.814610 (PMC8893279; doi:10.3389/fcvm.2022.814610)
Supplement: Supplementary file 1 [file Table_1.DOCX]

Supplementary Material

**Supplemental Table 1: Online questions used to confirm self-reported cardiovascular diseases**

A. Myocardial infarction:

1. Has a doctor ever told you that you had a heart attack or myocardial infarction?

2. Were you hospitalized for this heart attack?

3. Did you have a cardiac catheterization or a coronary angiogram?

B. Stroke:

1. Have you ever experienced any symptoms of sudden weakness or sudden loss of vision, particularly in one eye or sudden inability to talk or understand speech or sudden unexplained dizziness or unsteadiness that doesn’t go away or sudden severe headaches with no known cause?

2. Has your doctor ever told you that you had a stroke?

3. Were you ever hospitalized for treatment of a stroke?

C. Atrial fibrillation:

1. Has a doctor ever told you that you have atrial fibrillation?

D. Systolic heart failure/cardiomyopathy:

1. Have you ever been told that you have heart failure or congestive heart failure or CHF?

2. Have you ever been told that you had extra fluid in your lungs and/or legs and ankles that was treated with a diuretic medicine?

3. Have you ever been hospitalized for treatment for extra fluid in your lungs and/or legs and ankles?

E. Aortic dissection:

1. Have you ever been told you have had an aortic dissection?

2. Have you ever had or been told you have a rip or tear in your aorta?

3. Have you ever been hospitalized for a rip or tear in your aorta?
